# Measuring and improving the quality of tuberculosis care: A framework and implications from the *Lancet Global Health* Commission

**DOI:** 10.1016/j.jctube.2019.100112

**Published:** 2019-06-19

**Authors:** Catherine Arsenault, Sanam Roder-DeWan, Margaret E. Kruk

**Affiliations:** a Department of Global Health and Population, Harvard T.H. Chan School of Public Health, 665 Huntington Ave., Building 1, 1115, Boston, MA 02115, United States; b Ifakara Health Institute, Kiko Ave, Dar es Salaam, Tanzania

**Keywords:** Developing countries, Health systems, Quality, Measurement, Monitoring, Improvement

## Abstract

In this article, we describe the framework of the *Lancet Global Health* Commission on High Quality Health Systems, propose new and undermeasured indicators of TB care quality, and discuss implications of the Commission's key conclusions for measuring and improving the quality of TB care services. The Commission contends that measurement of quality should focus on the processes of care and their impacts. In addition to monitoring treatment coverage and the availability of tools, governments should consider indicators of clinical competence (for e.g. ability of providers to correctly diagnose TB and adhere to treatment guidelines), of timely, continuous and integrated care and of respectful and patient-centered care. Indicators of impact include TB mortality and treatment success rates, but also quality of life and daily functioning among TB patients, public trust in TB services, and bypassing of the formal health system for TB care. Cascades of care, from initial care seeking to recurrence-free survival, should be built in every high-burden country to monitor quality longitudinally. In turn, improvement efforts should target the foundations of health systems and consider the Commission's four universal actions: governing for quality, redesigning service delivery, transforming the health workforce and igniting demand for quality TB services. Important work remains to validate new indicators of TB care quality, develop data collection systems for new measures, and to test new strategies for improving the delivery of competent and respectful TB care.

## 1 Introduction

Tuberculosis (TB) experts are increasingly acknowledging that expanding diagnosis and treatment coverage alone will not suffice for “building a TB-free world”, and that high-quality health systems are essential [1]. After an individual develops active TB, they must navigate a long and complex process of care-seeking, diagnosis, linkage to care, treatment initiation, notification to national TB programs, and follow-up [2]. TB is therefore a condition that is particularly sensitive to the quality of health systems. In addition, TB is preventable, treatable and curable. Nonetheless, millions continue to die from the condition every year.

A recent study estimated that half of TB deaths in 2016 were due to poor-quality care while the other half resulted from non-utilization of the health system [3]. Poor-quality care is now an equal barrier to reducing TB mortality than insufficient access to care.

What is a high-quality health system? In 2018, three separate reports aimed to answer this questions and to identify approaches to redressing global inequalities in health care quality [4]. The *Lancet Global Health* Commission on High Quality Health Systems – a group of 30 academics, policymakers, and health system stakeholders from 18 countries – proposed a new definition and framework for high-quality health systems, described available data on quality in low- and middle-income countries (LMICs), and provided recommendations for quality measurement and improvement [5]. Improving the quality of TB services first requires that it is adequately defined, measured and monitored. The Commission's recommendations and framework were developed for understanding quality throughout the health system, for all health needs and across all health system platforms (community outreach, primary and tertiary care). In this paper, we describe the *Lancet Global Health* Commission's framework and discuss implications of the Commission's key messages in the context of TB. TB experts may use and adapt the framework and recommendations for exploring new approaches for measuring and improving TB.

## 2 High quality health system framework

**Fig. 1:**
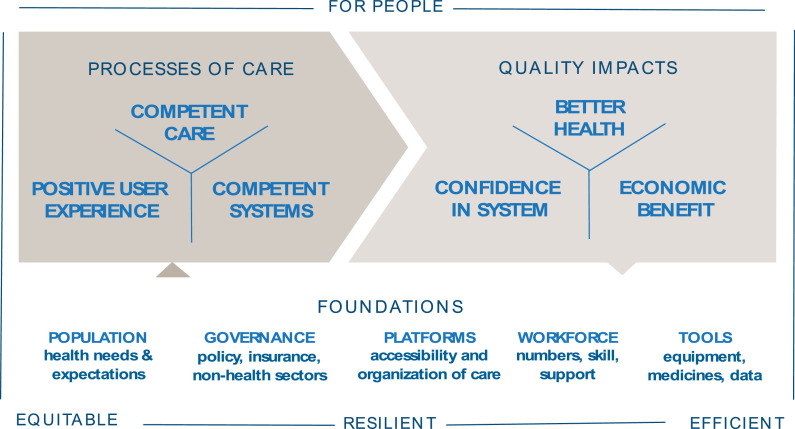
High-quality health system framework.

The Commission's framework on high-quality health systems shown in Fig. 1, emerged from a review of past work on health care quality and quality improvement, and from a recognition of the need to update the definition of high-quality health systems in light of today's health challenges, patient expectations and rising ambitions [5], [6], [7], [8]. First, the framework is underpinned by four values: high-quality health systems are for people, and they are equitable, resilient, and efficient. The emphasis on people-centeredness and equity is especially crucial for a highly stigmatized conditions such as TB that predominantly affects vulnerable social groups. The framework is centered around three key domains: health system foundations, processes of care and quality impacts. The Commission argued that quality should be primarily measured based on the processes of care and their impacts. In turn, health system foundations should be targeted by improvement strategies. Below, we define these three domains and discuss their implications for measuring and improving the quality of TB services. Readers can refer to the Commission report Sections 1 and 2 for more detailed definitions of the framework dimensions, and Sections 4 and 5 for additional material on measuring and improving health care quality.

## 3 Quality impacts

High quality health systems should produce better health, they should garner people's confidence and trust, and should produce economic benefits for people and countries.

### 3.1 Health

Mortality, morbidity and patient-reported outcomes measures (PROMs) (e.g. function, symptoms, pain, wellbeing) are useful to monitor the impact of health services on health. Half of TB deaths in 2016 were among people who accessed the health system [3]. Given that 95% of TB deaths are avoidable, the number of people who die from TB is an essential indicator of health system quality [1]. Nonetheless, mortality alone does not capture the full burden of poor-quality TB care. For many people in LMICs, access to care does not result in the control of this manageable condition. Treatment success/failure rates are also reflective of the quality of TB care. TB is also a disease that can seriously undermine the quality of life (QOL) in people who suffer from it [9]. For example, TB and drug-resistant TB are associated with an important proportion of the global burden of serious health-related suffering [10]. QOL is increasingly used to evaluate patient outcomes rather than traditional criteria such as mortality and illness [9]. Improved daily functioning and QOL among TB patients should be considered to monitor quality of TB services.

### 3.2 Confidence

The Commission also argued that the population's confidence in the health system reflects the quality of care available to them and is an important indicator of health system performance. For example, the Commission found that few people believe their health system works well in LMICs (only 24% across 11 LMICs), while the proportion of people who trusted their health system was double in high-income countries (48% across 11 high-income countries) [5]. People who do not trust their health system will often choose to forgo care for symptoms of TB, leading to long diagnostic delays and disease transmission. They are also less likely to be retained in care and to adhere to treatments contributing to the rise in MDR/XDR TB. Poor confidence may also lead to bypassing of the public or formal health system for the private or informal sector. The recent *Lancet* Commission on TB revealed that as many as 50%−60% of TB patients first seek care in ayurvedic or homeopathic doctors, pharmacists and private sectors [1]. The private sector often includes a heterogeneous mix of highly qualified providers (treating the richest segment of the population) and highly unqualified providers and facilities [11]. Confidence and public trust have rarely been measured in LMICs but is routinely collected in high-income countries [12]. Monitoring trust in the formal health system over time and for specific conditions such as TB, could be useful to inform policy decisions. For example, monitoring public trust among the population as a whole, across regions and over time, may help target efforts and monitor improvements in quality.

### 3.3 Economic benefits

The *Lancet Global Health* Commission described three types of economic consequences that could be averted by high-quality health systems: the economic effects of premature mortality, health system waste, and catastrophic or impoverishing health expenditures faced by households. A study estimated that avoidable TB deaths in twenty-two high-burden countries resulted in $3.2 trillion in welfare losses over a decade [13]. The *Lancet* Commission on TB also estimated that the value of the benefits of averting a death from TB exceeds the value of its costs by a factor of 3 to 5 [1]. Health system waste due to poor-quality TB care includes the overuse of harmful medicine such as steroids and fluoroquinolones (which can hide symptoms and delay diagnosis) and hospitalizations due to TB that could be avoided by high-quality primary care [14], [15]. In the case of TB, inappropriate treatments are particularly costly as they contribute to drug resistance and continued transmission of TB, in addition to their unnecessary out-of-pocket costs for the patient [16]. The cost of treating MDR/XDR TB strains can be 9–25 times higher than treating drug-susceptible TB [17]. Finally, TB patients risk suffering from catastrophic or impoverishing expenditures when repeating visits of poor-quality services or when forced to seek care from costly providers. Ensuring that everyone can access care without risking catastrophic medical costs is an integral component of a high-quality health system.

## 4 Processes of care

The second domain of the framework relates to the quality of the processes of care. The quality of the processes of care is reflected by the levels of competent, respectful, patient-centered care and requires that competent systems are in place to support health care providers.

### 4.1 Competent care and systems

Competent care means that all TB patients should be managed according to the latest evidence-based guidance for TB care. Standardized patients’ studies have shown that many providers in high-burden countries do not follow the International Standards for TB Care guidelines [18], [19]. Competent care also requires diagnostic accuracy and immediate treatment. In LMICs, many providers fail to diagnose TB further delaying treatment. Across six sub-Saharan African countries, diagnostic accuracy from clinical vignettes ranged from only 52% of providers correctly identifying TB in Ethiopia, to 86% of providers in Tanzania [5].

**Fig. 2:**
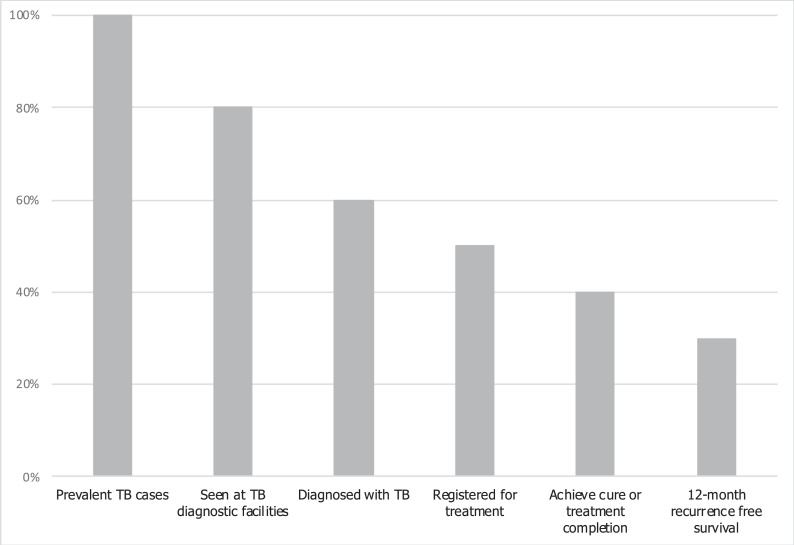
Hypothetical national TB cascade of care.

A heavy reliance on outdated diagnostic technologies also contributes to misdiagnoses: many countries continue to rely on often inaccurate smear microscopy [20], [21]. In turn, health systems must ensure patient safety, appropriate prevention and detection, case notification, timely treatment, and continuous and integrated of care. Early case detection and timely treatment is fundamental to interrupt TB progression and transmission. TB screening among high-risk groups is a recommended strategy and an essential function of high-quality health systems [22]. Continuity of care is reflected by the ability of health systems to retain people in care, coordinate care overtime and ensure adequate follow-up. Cascades of care can be particularly useful tools to monitor continuity of care (Fig. 2) [23], [24]. Studies in India and South Africa have shown that patients are lost at every steps of the cascade: many TB-affected people reach facilities but are never diagnosed, some are diagnosed but never start treatment, others start treatment but never complete it [23], [24]. Integration of care is the extent to which health services are delivered in a complementary manner. This means when seeking care for TB, people's other health needs (e.g. HIV, diabetes or pregnancy) should be addressed by health systems in a coherent and patient-centered manner.

### 4.2 User experience

An important, and vastly under-measured dimension of quality, relates to the user's (or patient's) experience with care in terms of respect and patient-centeredness. Respectful care includes respect for patient privacy, confidentiality and dignity and a caring and compassionate attitude of staff. A positive user experience also requires that TB services are user-focused i.e. that they are as easy as possible to use, accessible, affordable and in accordance with patient values. User experience is infrequently assessed in LMIC health systems and more research is needed to determine best indicators and measurement approaches to monitor user experience with TB services. TB programs should consider this crucial dimension of quality. In addition to its intrinsic value, a positive user experience can improve retention in care, adherence to treatment and overall confidence in the health system, likely leading to better health outcomes [5]. Ensuring patient-centered care is a particular challenge for TB, given the long treatment plans required for detecting and curing the condition. Nonetheless, it remains crucial to achieving good health outcomes.

## 5 Measuring TB quality

We reviewed the indicators currently used for global and national monitoring of the performance of TB programs [22], [25], [26]. Existing indicators such as TB death rates and treatment success rates reflect the impact of health system quality on health outcomes. The World Health Organization's Global TB report also includes an indicator for the proportion of TB patients facing catastrophic costs [26]. Case detection and notification rates and treatment default rates reflect the ability of health systems to detect, follow-up and retain people in care. These are important indicators of health system quality. Most of the other indicators currently used for TB relate to treatment coverage or focus on the availability of inputs necessary for care (e.g. laboratories, drugs, human resources, presence of TB policies). Crucial dimensions of the processes and impacts of TB care fail to be captured in current measurement including the clinical competence of TB care providers (e.g. ability of providers to correctly diagnose TB and adhere to treatment guidelines), the quality of user experience, patient satisfaction and TB morbidity (e.g. PROMs, persistent symptoms, quality of life).

In Table 1, we propose new indicators of TB care quality that should be considered for national monitoring of the quality TB care. New indicators are followed by an asterisk while existing indicators are in black. Some of these new indicators remain to be tested in the context of TB. However, they have been either previously used in TB studies, or for other health conditions, largely high-income country contexts [12], [27], [28], [29], [30].

**Table 1:** Dimensions of quality and illustrative indicators to monitor the quality of TB care at national levels.

Quality impacts	
Health	- Avertable TB deaths
	- TB treatment success rate
	- Daily functioning and quality of life among TB patients [9]^⁎^
	- Serious health-related suffering caused by TB [10]^⁎^
Confidence	- Proportion of TB patients who bypassed the public system for care^⁎^
	- Proportion of TB patients who are confident in their ability to receive the most effective treatment if they are sick [12]^⁎^
	- Proportion of TB patients who would recommend the clinic to others with the disease^⁎^
Economic	- Number of productive days lost to TB^⁎^
	- Proportion of TB patients with catastrophic care expenditures
	- Avoidable hospitalizations due to TB^⁎^
**Processes of care**	
Competent care	- Proportion of providers correctly diagnosing TB^⁎^
	- Proportion of patients managed according to the International Standards for TB Care guidelines^⁎^
Competent systems	- Proportion of high-risk individuals screened for TB
	- TB case detection rate
	- TB case notification rate
	- Average days between first contact with the health system and definitive TB diagnosis and treatment [31]^⁎^
	- National TB cascades of care (showing the proportion of patients lost at every step) (Fig. 2) [23], [24]
User experience	- Proportion of TB patients with high ratings for provider's respectful attitude, communication, explanations received, respect for their privacy and confidentiality^⁎^
	- Average wait time in TB diagnostic centers^⁎^

### 5.1 Data sources

Research is needed to carefully validate new indicators of quality and to identify data collection methods. While existing data sources such as vital registries and routine health information systems can provide some of the data needed to measure the indicators in Table 1, new data will also be needed. We describe some of the data collection methods previously employed to measure quality. For example, direct clinical observations – where a trained observer records performance during a clinical visit typically using a checklist – have been used to assess clinical practice for primary care and labor and delivery services in several countries [32], [33]. Studies on the quality of TB care have also pioneered the use of the standardized-patient method to measure clinical practice [2], [18], [19], [34], [35]. Standardized patients, also known as mystery patients, involves training individuals to portray a patient with symptoms of TB, and having them record the clinical decisions while the provider is blinded to the assessment. The TB field has also relied on modeling approaches to estimate the performance of TB control programs in high-burden countries [23], [36], [37]. Others have used provider vignettes (knowledge tests) to assess provider knowledge as a proxy for clinical competence [38]. Other data sources for quality include client exit interviews or population surveys (for example, to measure user experience, confidence or PROMs), register abstraction or review of medical records. The different strengths and limitations of these methods are described in the Commission's appendix [5]. Cohort studies may also be needed to assess clinical quality longitudinally and build cascades of care for TB from initial care-seeking to recurrence-free survival (Fig. 2) [23]. As mentioned previously, cascades of care can be particularly useful tools to visualize quality longitudinally and across health system platforms. However, determining how to collect data from individuals at each step of the cascade at national levels remains a challenge. Further research is needed to develop data collection systems for these undermeasured indicators of quality.

## 6 Health system foundations

Health system foundations (Fig. 1) begin with the population, including people who suffer from TB, their health needs, knowledge and preferences. Governance includes financing, policies and accountability mechanisms. Health system platforms include the number of facilities and their distribution and organization, and the systems connecting the different levels of care. Providers, from health workers to managers, and their knowledge, skills and training, constitute another crucial foundation. Finally, health systems require physical tools such as equipment, diagnostics, vaccines, medicines, supplies and information systems. Although these foundations are crucial to the provision of high-quality TB care, their sole presence does not guarantee good health outcomes or that good quality care is provided to people [39]. For example, availability of TB diagnostic tools in a facility might not mean availability when needed, and that they are used for the right people, at the right time [32]. Measurement of health system foundations, ideally in real-time, is important to manage the health system, for example to manage drug stocks and provider numbers. However, foundation measures are not useful measures of health system quality at national levels as they may not reflect actual health system performance for patients.

That said, foundations of health systems are the most appropriate starting point for improvement. This recommendation stands in contrast to standard approaches to quality improvement which targets individual facilities or provider behavior. Recent evidence shows that in-service training and other interventions targeted at the point of care, mainstays of quality improvement, have only a modest effect on quality of services delivered [40]. The Commission contends that without reforms to strengthen health systems, facility-level quality improvement schemes are unlikely to create sustainable impact at the scale necessary to overcome large quality gaps evident in TB care in LMICs today [5]. We have seen this with TB diagnostics, where availability of new tools is difficult to translate into adoption at scale because of barriers in underlying system performance [41]. The Commission recommends investing in the foundations of health systems by considering four universal actions for improving quality: 1- governing for quality, 2- redesigning service delivery, 3- transforming the health workforce and 4- igniting people's demand for high-quality care.

### 6.1 Universal action 1: governing for quality

Governing for quality means refocusing the national lens on *effective* coverage of TB care, rather than treatment coverage alone. Political leaders, with the support of technical experts, must express a commitment to quality and align national policy, strategy and implementation around a vision for high-quality services. For example, countries can govern for the quality of TB care by considering losses-to-follow-up and treatment failures as core *health system* responsibilities rather than facility-level shortcomings. This high-level leadership translates into improvement through tougher regulations that keep system actors accountable for delivering high quality care. These regulations should be based on international standards for TB care that are well-supported by evidence, but should also be adapted and endorsed by national organizations. Bringing civil society organization, such as professional organizations, into the dialogue on quality can be especially important for ensuring quality in the private sector where oversight and accountability can be particularly challenging [42]. Finally, development partners have a role to play in making quality governance possible; short cycles of vertical programming for TB make critical improvements such as integration into primary care very difficult [43]. Governing for quality raises the national standard for TB care by measuring success differently; high coverage of poor-quality care is no longer an acceptable outcome.

### 6.2 Universal action 2: redesigning service delivery

Redesigning service delivery in the context of TB care is critical for TB, but also for making high-quality healthcare of other conditions possible. Redesign starts with a critical analysis of where and by whom TB services are being delivered, including the contribution of the private sector. Redesign also requires an understanding of patterns and barriers for individuals with TB symptoms to seek and access diagnosis and treatment. To restructure these systems and maximize quality for TB, this review cannot be limited to TB. For example, routine TB care, including active case finding, should be delivered at the primary care level where personnel are more likely to understand local social networks and practices. More complicated cases should be managed at secondary or tertiary care facilities; many countries are moving towards this model of community based care [44]. However, health systems will need to consider moving other less complex conditions, such as routine hypertension, out of secondary and tertiary facilities to decongest and make resources available for patients that need them. Redesign efforts will need to be particularly sensitive to how changes may impact access, equity and quality for vulnerable groups and will need to be informed by local data on quality and disease patterns. For example, a study in China showed that integrating TB care into primary care could actually compromise population detection and treatment in rural areas due to poor quality of care in distal health facilities. Redesigning in rural China would need to carefully address this barrier before restructuring the system [34].

### 6.3 Universal action 3: transform the health workforce

The Commission recommends that improvement efforts should target pre-service training such that providers enter practice with better baseline knowledge and skills. For TB, this means learning how to diagnose and manage latent and active TB, but it also means graduating with demonstrable competence in delivering TB care. Gaps between provider knowledge and the actual care that they deliver in clinical settings can be large; measuring and improving knowledge alone is unlikely to have large impacts [45]. In order to build a workforce that is able to prevent and treat a complex, socially stigmatized disease such as TB, clinical education will need to include broader (and softer) skills such as population health management, patient counseling, cultural sensitivity, and understanding bias. Providers should enter practice with a solid foundation in ethical practice and in patient-centered respectful care. These are not skills that can be gained in day-long trainings. Rather, they must be introduced early and modeled by respected clinical educators: they must be woven into the core of how providers work.

### 6.4 Universal action 4: ignite demand for quality

Igniting demand for quality means challenging countries and partners to raise population expectations for quality care. To do so, health systems will need to share information and educate communities about what constitute good quality care and create opportunities for meaningful participation. Appropriate expectations of healthcare quality and demand for high quality services can put pressure on systems to deliver effective care. We have seen effective advocacy around TB drug access already; for example, advocates in South Africa have played a key role in the country's adoption of bedaquiline for multi-drug resistant TB. This type of action is a health system resource that needs to be fostered and supported. Improving patient education is also critical to ensure that systems are used appropriately. Efforts to integrate TB services with primary care, especially in middle-income countries, has been challenging in part due to the perception that specialty care was *always* better for TB [46].

## 7 Conclusion

Further research is needed to define and validate new indicators of TB care quality and to develop data collection systems for these undermeasured dimensions in high-burden countries. New technologies should be explored to improve data accuracy, timely data collection and reduce measurement burden (e.g. phone/SMS surveys, wearable trackers etc.). Countries and global partners should invest in data systems for quality. The Commission's four universal actions should also be considered and tested for improving the delivery of competent and respectful TB care.

## Funding sources

This work was supported by the Bill & Melinda Gates Foundation (Grant no. OPP1161450), Seattle, WA.

## Ethical statement

The authors have no conflicts of interest

## CRediT authorship contribution statement

**Catherine Arsenault:** Conceptualization, Writing - original draft, Validation. **Sanam Roder-DeWan:** Writing - original draft, Validation. **Margaret E. Kruk:** Conceptualization, Writing - review & editing, Validation.

## Appendix Supplementary materials

Image, application 1
